# Risk of Peripheral Artery Occlusive Disease in Patients with Vertigo, Tinnitus, or Sudden Deafness: A Secondary Case-Control Analysis of a Nationwide, Population-Based Health Claims Database

**DOI:** 10.1371/journal.pone.0162629

**Published:** 2016-09-15

**Authors:** Malcolm Koo, Jin-Cherng Chen, Juen-Haur Hwang

**Affiliations:** 1 Department of Medical Research, Dalin Tzu Chi Hospital, Buddhist Tzu Chi Medical Foundation, Dalin, Chiayi, Taiwan; 2 Dalla Lana School of Public Health, University of Toronto, Toronto, Ontario, Canada; 3 Department of Neurosurgery, Dalin Tzu Chi Hospital, Buddhist Tzu Chi Medical Foundation, Dalin, Chiayi, Taiwan; 4 School of Medicine, Tzu Chi University, Hualien, Taiwan; 5 Department of Otolaryngology, Dalin Tzu Chi Hospital, Buddhist Tzu Chi Medical Foundation, Dalin, Chiayi, Taiwan; 6 Sleep Center, Dalin Tzu Chi Hospital, Buddhist Tzu Chi Medical Foundation, Dalin, Chiayi, Taiwan; Medical University Innsbruck, AUSTRIA

## Abstract

**Background:**

Cochleovestibular symptoms, such as vertigo, tinnitus, and sudden deafness, are common manifestations of microvascular diseases. However, it is unclear whether these symptoms occurred preceding the diagnosis of peripheral artery occlusive disease (PAOD). Therefore, the aim of this case-control study was to investigate the risk of PAOD among patients with vertigo, tinnitus, and sudden deafness using a nationwide, population-based health claim database in Taiwan.

**Methods:**

We identified 5,340 adult patients with PAOD diagnosed between January 1, 2006 and December 31, 2010 and 16,020 controls, frequency matched on age interval, sex, and year of index date, from the Taiwan National Health Insurance Research Database. Risks of PAOD in patients with vertigo, tinnitus, or sudden deafness were separately evaluated with multivariate logistic regression analyses.

**Results:**

Of the 5,340 patients with PAOD, 12.7%, 6.7%, and 0.3% were diagnosed with vertigo, tinnitus, and sudden deafness, respectively. In the controls, 10.6%, 6.1%, and 0.3% were diagnosed with vertigo (P < 0.001), tinnitus (P = 0.161), and sudden deafness (P = 0.774), respectively. Results from the multivariate logistic regression analyses showed that the risk of PAOD was significantly increased in patients with vertigo (adjusted odds ratio = 1.12, P = 0.027) but not in those with tinnitus or sudden deafness.

**Conclusions:**

A modest increase in the risk of PAOD was observed among Taiwanese patients with vertigo, after adjustment for comorbidities.

## Introduction

Peripheral artery occlusive disease (PAOD) is a chronic disease due to obstruction of the small or medium sized arteries that provide blood supply to various body organs, especially the lower extremities. The prevalence of PAOD increased gradually with age and it was estimated to be 1.5% among individuals in their forties and increased to 27% in those 80 years and over [1]. In the western world, PAOD represents a leading cause of morbidity associated with intermittent claudication and an increased risk of mortality as a result of coexistent coronary artery and cerebrovascular atherosclerosis [2]. It was estimated that US$4.37 billion was spent in the United States on PAOD-related treatment with 88% of the expenditures associated with inpatient care [3].

PAOD is a complex disease with a number of risk factors, including both environmental and genetic [4]. Major cardiovascular disease (CVD) risk factors include diabetes, hypertension, dyslipidemia, and cigarette smoking [1]. Black ethnicity was also found to be a risk factor for PAOD, independent of diabetes, hypertension, or other CVD risk factors [5]. In addition, previous studies have suggested that certain microvascular diseases, notably retinopathy and nephropathy, precede the occurrence of macrovascular diseases. In a multiethnic population-based study of 6,147 adults, retinopathy was found to be significantly associated with a moderate-to-severe coronary artery calcification score, with an odds ratio (OR) of 1.43, after adjustment for age, sex, ethnicity, blood pressure, diabetes, lipid profile, smoking, and other risk factors [6]. This finding supports the concept that shared pathophysiologic processes might contribute to both microvascular and macrovascular diseases. In addition, a systematic review of 25 studies with 54,117 individuals concluded that microvascular complications appeared to be associated with cardiovascular events. In particular, diabetic retinopathy was associated with 1.7-fold increased risk for cardiovascular events while albuminuria or reduced glomerular filtration rate was associated with a 2-fold increased risk for cardiovascular events, after adjustment for conventional cardiovascular risk factors, diabetes duration, and glycemic control [7].

Cochleovestibular symptoms, such as vertigo, tinnitus, and sudden deafness, are common manifestations of microvascular diseases. However, it is still unclear whether these symptoms could increase the risk of PAOD. Thus, we investigated the risk of PAOD in patients diagnosed with vertigo, tinnitus, or sudden deafness using data based on a nationwide, population-based health claim database.

## Methods

This study was approved by the institutional review board of the Dalin Tzu Chi Hospital, Buddhist Tzu Chi Medical Foundation, Taiwan (No. B10202021). Since the NHIRD files contain only de-identified secondary data, the review board waived the requirement for obtaining informed consent from the patients.

### Study design and data source

This nationwide, population-based case-control study analyzed the data obtained from the Longitudinal Health Insurance Database 2000 (LHID 2000), which is a subset of the Taiwan National Health Insurance Research Database (NHIRD). The LHID 2000 contains medical services utilization information from 1996 to 2010 for a randomly selected sample of one million beneficiaries registered in 2000, representing approximately 5% of Taiwan’s population. The Taiwan National Health Insurance is a universal single-payer compulsory health insurance program instituted in March 1995. As of the end of 2011, about 99% of Taiwan’s population was enrolled in the program. Under the insurance plan, enrollees can receive almost free access to healthcare, including inpatient and ambulatory care, except for a small copayment of US$1.7 for a visit to a clinic [8].

### Study sample and measurements

**Fig 1:**
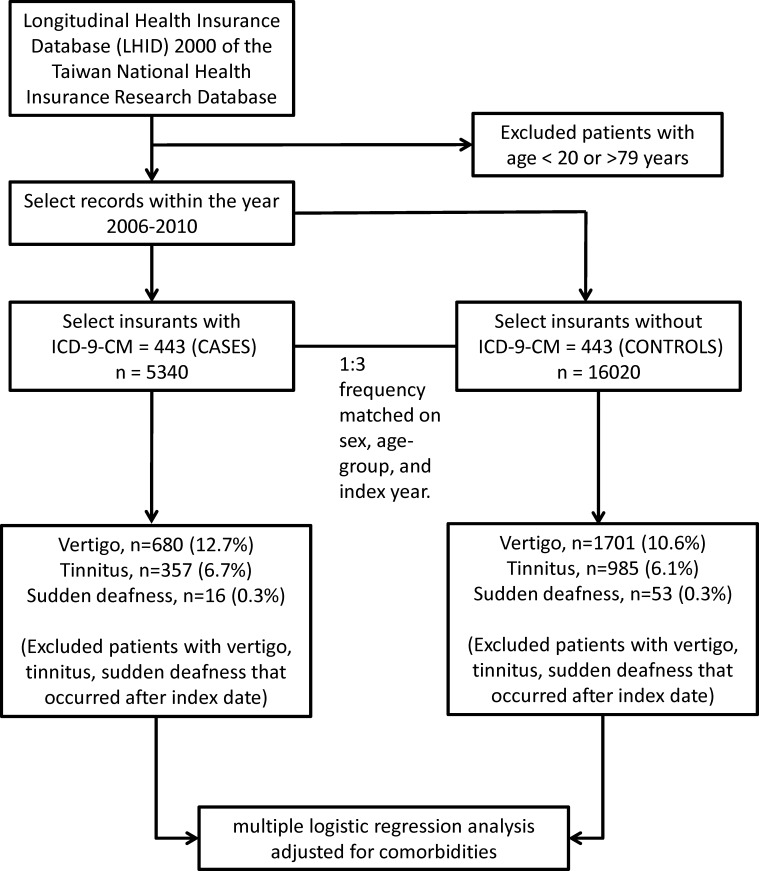
Flow diagram of study design. ICD-9-CM: International Classification of Diseases, 9^th^ Revision, Clinical Modification.

Cases were defined as patients diagnosed with PAOD, based on the International Classification of Diseases, 9^th^ Revision, Clinical Modification (ICD-9-CM) code 443 that occurred between January 1, 2006 and December 31, 2010. Three controls per case were randomly selected from the LHID 2000, frequency matched by sex, age interval (20–39, 40–59, and 60–79 years), and year of index date. Patients under the age of 20 or over 79 years were excluded (Fig 1).

Diagnoses of vertigo (ICD-9-CM codes 386.1, 386.2, 386.5, 386.8, and 386.9), tinnitus (ICD-9-CM code 388.3), and sudden deafness (ICD-9-CM code 388.2) in the cases and controls prior to the index date were assessed. Other comorbidities included hypertension (ICD-9-CM codes 401–405), diabetes mellitus (DM) (ICD-9-CM code 250), coronary artery disease (CAD) or myocardial infarction (MI) (ICD-9-CM codes 414, 410, and 429), chronic kidney disease (CKD) (ICD-9-CM codes 585 and 586), hyperlipidemia (ICD-9-CM code 272), hyperuricemia (ICD-9-CM codes 274 and 790.6), and ischemic cerebrovascular diseases (ICVD) (ICD-9-CM codes 433.01, 433.10, 433.11, 433.21, 433.90, 434.xx, 435.1, 435.3, 435.8, 435.9, 436, 437.0. 437.8, 437.9, 438.89, and 438.9) were also assessed.

### Statistical analysis

Distributions of age intervals, sex, vertigo, tinnitus, sudden deafness, and comorbidities between patient with PAOD and controls were evaluated using Chi-square test or Fisher’s exact test, as appropriate. Unconditional multivariate logistic regression analyses were used to evaluate the risks of PAOD associated with vertigo, tinnitus, or sudden deafness, adjusting for comorbidities. All analyses were performed using IBM SPSS Statistics software package, version 21.0 (IBM Corp., Armonk, NY, USA). A P < 0.05 was considered statistically significant.

## Results

Table 1 shows the distribution of age intervals, sex, vertigo, tinnitus, sudden deafness, and selected comorbidities for the 5,340 patients with PAOD and the 16,020 controls. There were no significant differences in the distribution of age interval and sex as a result of the frequency matching on these two variables. Among the patients with PAOD, 12.7%, 6.7%, and 0.3% were diagnosed with vertigo, tinnitus, and sudden deafness, respectively. Among controls, 10.6% were diagnosed with vertigo (P < 0.001), 6.1% were diagnosed with tinnitus (P = 0.161), and 0.3% were diagnosed with sudden deafness (P = 0.774). To assess the independent effects of vertigo, tinnitus, and sudden deafness, that is, when they existed as isolated symptoms, we created three additional variables, namely, “vertigo but no tinnitus or sudden deafness”, “tinnitus but no vertigo or sudden deafness”, and “sudden deafness but no vertigo or tinnitus”. The proportion of PAOD (9.9%) among the patients with vertigo but no tinnitus or sudden deafness was significantly higher (P < 0.001) compared with that of the controls (8.1%). There were no significant differences in the distribution of tinnitus but no vertigo or sudden deafness (P = 0.645) or sudden deafness but no vertigo or tinnitus (P > 0.999) between patients with PAOD and controls. Furthermore, the cases had a significantly higher frequency of comorbid conditions, including hypertension, DM, CAD or MI, CKD, hyperlipidemia, hyperuricemia, and ICVD, compared with controls.

**Table 1:** Basic characteristics of patients with peripheral artery occlusive disease and controls.

Variable		n (%)		*P* value
		PAOD	Controls	
		(*n* = 5,340)	(*n* = 16,020)	
Age interval (years)				> 0.999
	20–39	512 (9.6)	1,536 (9.6)	
	40–59	2,162 (40.5)	6,486 (40.5)	
	60–79	2,666 (49.9)	7,998 (49.9)	
Sex				> 0.999
	male	2,261 (42.3)	6,783 (42.3)	
	female	3,079 (57.7)	9,237 (57.7)	
Vertigo				< 0.001
	yes	680 (12.7)	1,701 (10.6)	
	no	4,660 (87.3)	14,319 (89.4)	
Tinnitus				0.161
	yes	357 (6.7)	985 (6.1)	
	no	4,983 (93.3)	15,035 (93.9)	
Sudden deafness				0.774
	yes	16 (0.3)	53 (0.3)	
	no	5,324 (99.7)	15,967 (99.7)	
Vertigo but no tinnitus or sudden deafness				< 0.001
	yes	527 (9.9)	1,302 (8.1)	
	no	4,813 (90.1)	14,718 (91.9)	
Tinnitus but no vertigo or sudden deafness				0.645
	yes	200 (3.7)	578 (3.6)	
	no	5,140 (96.3)	15,442 (96.4)	
Sudden deafness but no vertigo or tinnitus				> 0.999
	yes	2 (0.04)	8 (0.05)	
	no	5,338 (99.96)	16,012 (99.95)	
Hypertension				< 0.001
	yes	2,722 (51.0)	6,967 (43.5)	
	no	2,618 (49.0)	9,053 (56.5)	
Diabetes mellitus				< 0.001
	yes	1,547 (29.0)	3,606 (22.5)	
	no	3,793 (71.0)	12,414 (77.5)	
CAD or myocardial infarction				< 0.001
	yes	1,050 (19.7)	2,342 (14.6)	
	no	4,290 (80.3)	13,678 (85.4)	
Chronic kidney disease				< 0.001
	yes	313 (5.9)	523 (3.3)	
	no	5,027 (94.1)	15,497 (96.7)	
Hyperlipidemia				< 0.001
	yes	1,810 (33.9)	4,329 (27.0)	
	no	3,530 (66.1)	11,691 (73.0)	
Hyperuricemia				< 0.001
	yes	853 (16.0)	1,951 (12.2)	
	no	4,487 (84.0)	14,069 (87.8)	
ICVD				< 0.001
	yes	488 (9.1)	1,175 (7.3)	
	no	4,852 (90.9)	14,845 (92.7)	

PAOD: peripheral artery occlusive disease, CAD: coronary artery disease, ICVD: ischemic cerebrovascular diseases.

Table 2 showed the results of multivariate logistic regression analyses of vertigo, tinnitus, and sudden deafness. After adjustment for hypertension, DM, CAD or MI, CKD, hyperlipidemia, hyperuricemia, and ICVD, vertigo remained significantly associated with the risk of PAOD (adjusted OR = 1.12, P = 0.027), whereas tinnitus and sudden deafness were not associated with PAOD. The three variables for assessing the isolated effects of vertigo, tinnitus, and sudden deafness showed similar patterns of association compared with the original variables. Only the variable vertigo but no tinnitus or sudden deafness showed a significant association with PAOD (adjusted OR = 1.13, P = 0.025).

**Table 2:** Multivariate logistic regression analysis of the risk of peripheral artery occlusive disease among patients with vertigo, tinnitus, or sudden deafness.

Variable	Adjusted OR^a^ (95% CI)	P value
Vertigo	1.12 (1.01–1.23)	0.027
Tinnitus	1.02 (0.90–1.15)	0.798
Sudden deafness	0.84 (0.48–1.47)	0.538
Vertigo but no tinnitus or sudden deafness	1.13 (1.02–1.26)	0.025
Tinnitus but no vertigo or sudden deafness	0.99 (0.84–1.17)	0.918
Sudden deafness but no vertigo or tinnitus	0.69 (0.14–3.26)	0.637

^a^Adjusted for hypertension, diabetes mellitus, coronary artery disease or myocardial infarction, chronic kidney disease, hyperlipidemia, hyperuricemia, and ischemic cerebrovascular diseases.

OR: odds ratio, 95% CI: 95% confidence interval.

Table 3 showed the results of multivariate logistic regression analysis of vertigo stratified by sex. The association between vertigo and PAOD was significant only in female patients (adjusted OR = 1.15, P = 0.025) but not in male patients (adjusted OR = 1.06, P = 0.497).

**Table 3:** Multivariate logistic regression analyses of peripheral artery occlusive disease among male and female patients with vertigo.

Variable	Male				Female			
	PAOD	Controls	Adjusted OR^a^	P value	PAOD	Controls	Adjusted OR	P value
	n (%)	n (%)	(95% CI)		n (%)	n (%)	(95% CI)	
Vertigo			1.06 (0.90–1.25)	0.497			1.15 (1.02–1.30)	0.025
yes	228 (10.1)	571 (8.4)			452 (14.7)	1,130 (12.2)		
no	2,033 (89.9)	6,212 (91.6)			2,627 (85.3)	8,107 (87.8)		
Hypertension			1.51 (1.27–1.78)	<0.001			1.56 (1.38–1.76)	<0.001
yes	1,236 (54.7)	3,071 (45.3)			1,486 (48.3)	3,896 (42.2)		
No	1,025 (45.3)	3,712 (54.7)			1,596 (51.7)	5,341 (57.8)		
Diabetes mellitus			1.05 (0.89–1.25)	0.560			1.10 (0.96–1.25)	0.162
yes	714 (31.6)	1,582 (23.3)			833 (27.1)	2,024 (21.9)		
no	1,547 (68.4)	5,201 (76.7)			2,246 (72.9)	7,213 (78.1)		
CAD or myocardial infarction				<0.001				
yes	492 (21.8)	1,060 (15.6)	1.71 (1.44–2.03)		558 (18.1)	1,282 (13.9)	1.45 (1.26–1.66)	<0.001
no	1,769 (78.2)	5,723 (84.4)			2,521 (81.9)	7,955 (86.1)		
Chronic kidney disease			1.15 (0.86–1.54)	0.337			0.58 (0.42–0.79)	0.001
yes	166 (7.3)	272 (4.0)			147 (4.8)	251 (2.7)		
no	2,095 (92.7)	6,511 (96.0)			2,932 (95.2)	8,986 (97.3)		
Hyperlipidemia			1.33 (1.13–1.57)	0.001			1.59 (1.41–1.79)	<0.001
yes	745 (33.0)	1,765 (26.0)			1,065 (34.6)	2,564 (27.8)		
no	1,516 (67.0)	5,018 (74.0)			2,014 (65.4)	6,673 (72.2)		
Hyperuricemia			1.33 (1.12–1.58)	0.001			1.09 (0.92–1.30)	0.321
yes	509 (22.5)	1,245 (18.4)			344 (11.2)	706 (7.6)		
no	1,752 (77.5)	5,538 (81.6)			2,735 (88.8)	8,531 (92.4)		
ICVD			2.38 (1.97–2.89)	<0.001			2.55 (2.15–3.02)	<0.001
yes	270 (11.9)	615 (9.1)			218 (7.1)	560 (6.1)		
no	1,991 (88.1)	6,168 (90.9)			2,861 (92.9)	8,677 (93.9)		

^a^The odds ratios were simultaneously adjusted for all the variables included in the table.

OR: odds ratio, 95% CI: 95% confidence interval. PAOD: peripheral artery occlusive disease, CAD: coronary artery disease, ICVD: ischemic cerebrovascular diseases.

## Discussion

In this nationwide, population-based case-control study, we found vertigo was significantly associated with an increased risk of PAOD, particularly among women, after adjusting for a number of comorbid conditions that are considered to be conventional risk factors for PAOD. On the other hand, tinnitus and sudden deafness were not significantly associated with PAOD. To our knowledge, this is the first study that showed an association of vertigo and PAOD using population-based data.

Microvascular and macrovascular complications have similar etiologic characteristics and might share similar pathogenic mechanisms such as accumulation of advanced glycation end products [9]. Previous studies of patients with type 2 diabetes showed that microvascular complications, particularly retinopathy or nephropathy, were linked to an increased risk for cardiovascular events. This finding suggested the underlying mechanisms of atherosclerosis are relevant to diabetic microvascular diseases. In addition, rheological mechanisms including increased blood viscosity, reduced red blood cell deformability, reduced blood flow, and increased platelet aggregability, were relevant to both microvascular and macrovascular disease in type 2 diabetes [7].

Because of the close proximity of the cochlea and vestibular labyrinth, both structures share the common blood supply and therefore, we anticipated that cochlear microvascular disorder should affect both the vestibular labyrinth as vertigo and the cochlea as tinnitus and/or sudden deafness. Sudden sensorineural hearing loss was often associated with tinnitus and vertigo [10]. Furthermore, a meta-analysis of 13 studies reported an overall pooled OR of 2.15 (95% confidence interval = 1.72–2.68) of hearing impairment among diabetic cases compared with nondiabetic controls [11]. Since the severity of hearing loss appeared to correlate with progression of disease as reflected by changes in serum creatinine, it has been suggested that sensorineural hearing loss in patients with diabetes was caused by microangiopathic disease in the inner ear [12]. Nevertheless, our study showed that the risk of PAOD was increased only among patients with vertigo but not with tinnitus or sudden deafness. There are a few possible explanations for our findings.

First, both the vestibular labyrinth and cochlea share the common blood supply and lymphatic spaces and therefore, disorders of the inner ear often affect both structures. Nevertheless, the labyrinthine circulation is an end circulation with minimal collaterals from the otic capsule, which makes the vestibular labyrinth particularly vulnerable to ischemia [13,14]. Since episodes of vertigo can be caused by transient ischemia to the labyrinth, the association between vertigo and PAOD might be a reflection of microvascular insufficiency of the labyrinth. It is possible that vertigo is a more sensitive indicator of a change of microvasculation in the inner ear than tinnitus or sudden deafness. When patients experience a severe attack of vertigo, they may not be aware of a transient or mild hearing loss and tinnitus that occurred at the same time and therefore, failed to report their occurrence.

Second, a double-blind retrospective cohort study reported that a significantly higher number of patients with vertebral artery abnormality and at least three stroke risk factors had complained of isolated positional vertigo compared with patients with normal vertebral artery [15]. In addition, diminished blood flow in the vertebrobasilar artery system, detected using magnetic resonance imaging, had been reported in studies of patients with vertigo [16]. Therefore, vertigo might be considered as an indicator of vertebrobasilar artery system abnormality, which is a part of the generalized atherosclerosis associated with PAOD. Given the increased risk of myocardial infarction and incident ischemic stroke associated with both symptomatic and asymptomatic PAOD patients [17], diagnostic assessments for PAOD may be appropriate for those who are at risk for vascular events when presenting with vertigo.

Regarding the findings with stratification by sex, the association between vertigo and PAOD was significant only in female patients. It is plausible that female patients were more likely to report vertigo compared with male patients. Perceived societal gender expectations have been suggested to lead to gender differences in symptom reporting [18]. Therefore, the association between vertigo and PAOD in male patients might have been attenuated by the presence of misclassification due to a lower likelihood of reporting the symptom. Nevertheless, whether the higher prevalence of vertigo in females, observed in both the literature [19,20] and in our study, was a result of gender difference in symptom reporting will require further investigation.

The main strengths of our study are its sample size and the population-based design. The large sample size provided considerable statistical power for detecting small differences between cases and controls. Diagnoses of vertigo, tinnitus, and sudden deafness were collected before that of PAOD and thus can minimize the possibilities of recall bias in typical case-control studies. Nevertheless, the presence of PAOD, vertigo, tinnitus, and sudden deafness in this study were all based on ICD-9-CM codes and therefore, our results should be interpreted within this context. In this study, a number of comorbidities were included in the multivariate model to adjust for their potential confounding effects. In addition to the typical risk factors for cardiovascular disease (including hypertension, DM, CAD or MI, CKD, hyperlipidemia, and hyperuricemia) [21], ICVD, which can significantly affect the cochleovestibular system, was also included in the multivariate model. The types of ICVD included were acute cerebrovascular disease (ICD-9-CM code 436), occlusion of cerebral arteries (ICD-9-CM codes 434.00, 434.01, 434.10, 434.11, 434.9, 434.90, and 434.91), other cerebrovascular disease (ICD-9-CM codes 437.8, 437.9, 438.89, and 438.9), transient cerebral ischemia (ICD-9-CM codes 435.8 and 435.9), vertebral artery syndrome (ICD-9-CM code 435.1), vertebrobasilar artery syndrome (ICD-9-CM code 435.3), and occlusion of basilar artery, carotid artery or precerebral arteries (ICD-9-CM codes 433.01, 433.10, 433.11, 433.21, and 433.90). Nevertheless, there is no information on lifestyle factors such as cigarette smoking or the severity of vertigo, tinnitus, and sudden deafness, which is a limitation common to all studies based on analyses of the NHIRD administrative database.

In conclusion, we found a modest increase in the risk of PAOD among patients with vertigo, even after adjusting for comorbidities. Given the high prevalence and recurrence rate of vertigo and its negative impact on the quality of life, its effects on the risk of PAOD warrant further investigation.
